# Neuroinflammation and fractalkine signaling in Alzheimer’s disease

**DOI:** 10.1186/s12974-019-1412-9

**Published:** 2019-02-11

**Authors:** Dylan J. Finneran, Kevin R. Nash

**Affiliations:** Department of Molecular Pharmacology and Physiology, Morsani College of Medicine, University of South Florida, 12901 Bruce B Downs Bvld, Tampa, FL 33612 USA

**Keywords:** Neurodegeneration, Neuroinflammation, Fractalkine (CX3CL1), CX3CR1, Microglia

## Abstract

Alzheimer’s disease (AD) is a progressive, neurodegenerative disorder, and the most common form of dementia. As the understanding of AD has progressed, it is now believed that AD is an amyloid-initiated tauopathy with neuroinflammation serving as the link between amyloid deposition, tau pathology, and neurodegeneration. As microglia are the main immune effectors in the central nervous system, they have been the focus of attention in studies investigating the neuroinflammatory component of AD. Therefore, recent work has focused on immunomodulators, which can alter microglial activation without suppressing activity, as potential therapeutics for AD. Fractalkine (CX3CL1; FKN), a unique chemokine with a one-to-one relationship with its receptor, signals through its cognate receptor (CX3CR1) to reduce expression of pro-inflammatory genes in activated microglia. Disrupting FKN signaling has opposing effects on the two hallmark pathologies of AD, but over-expressing a soluble FKN has been shown to reduce tau pathology while not altering amyloid pathology. Recently, differential signaling has been reported when comparing two cleavage variants of soluble FKN. These differential effects may explain recent studies reporting seemingly conflicting results regarding the effect of FKN over expression on AD pathologies.

## Background

In the central nervous system (CNS), fractalkine (CX3CL1; FKN)) is expressed predominantly on neurons, and its receptor (CX3CR1) is expressed solely on microglia [1]. Produced as a transmembrane protein with an N-terminal chemokine domain followed by a long, mucin-like stalk, FKN can signal as either a membrane-bound protein or be cleaved by several proteases to generate a soluble fragment [2–4]. Signaling by FKN has been shown to reduce expression of pro-inflammatory genes in stimulated microglia [5, 6]. Unlike other chemokines, fractalkine shares a one-to-one relationship with its receptor, allowing neurons to directly regulate microglial activity.

Research has shown a strong link between microglial activity and neurodegeneration in several neurodegenerative disorders. Given the ability of FKN to mediate microglial activation, it has been the subject of much interest. However, it appears as though the effects of FKN signaling is both context- and ligand-specific. For instance, disrupting FKN signaling has been shown to be beneficial in stroke and models of amyloid deposition [7–10] while detrimental in tauopathy and Parkinson’s disease (PD) models [11–13]. Reports from neuropathic pain and Alzheimer’s disease (AD) models have further shown that there may be differential signaling elicited by full-length vs soluble FKN [14–16]. Here, we focus on the effects of FKN signaling in AD models as well as the differential effects of full length FKN signaling as compared to soluble FKN signaling.

### Alzheimer’s disease

Alzheimer’s disease, a fatal neurodegenerative disorder and the most common form of dementia, is characterized by progressive cognitive decline and memory loss. Most AD cases are late-onset and sporadic with the greatest risk factor for acquiring AD being age. In adults greater than 85, the prevalence of AD is greater than one in three, which results in a serious financial burden on patients and caregivers. Currently, there are no treatments that halt or slow the progression of the disease, and the pharmacotherapies available to patients only provide symptomatic relief that does not address the underlying pathology [17].

The two pathological hallmarks of AD are an accumulation of misfolded proteins: extracellular amyloid beta and intracellular microtubule-associated protein tau (MAPT). These inclusions lead to severe brain atrophy and neurodegeneration in the hippocampus and cortex. The current view is that AD is an amyloid-driven tauopathy [18]. Amyloid precursor protein (APP) is cleaved in a sequential manner first by β-secretase followed by γ-secretase, resulting in the generation of amyloid-beta (Aβ) and its subsequent accumulation. It is believed that this amyloid deposition triggers tau hyperphosphorylation and aggregation, which eventually leads to neurodegeneration. This is supported by the fact that individuals with Down syndrome, who have a triplication of all or part of chromosome 21 on which the amyloid precursor protein encoding gene resides, often develop amyloid plaques and an AD-like phenotype at a young age. Furthermore, all known mutations that cause familial AD either increase the production of or alter the aggregation properties of amyloid beta [19]. As such, many human trials have focused on clearing amyloid beta aggregates or reducing the amyloid burden on the human brain as a potential therapeutic [20]. A body of work has studied this approach, but it has yet to bear fruit.

#### Neuroinflammation in Alzheimer’s disease

As research into AD has progressed, an addendum has been made to the amyloid cascade hypothesis which inserts neuroinflammation as the causal link between amyloid deposition, tau pathology, and neurodegeneration [18]. Support for this link comes from both human tissue and mouse models, in which activated microglia have been observed surrounding amyloid [21]. Using the microglial translocator protein (TSPO) ligand as a marker of microglial activation, longitudinal in vivo imaging of prodromal and AD cases have demonstrated that microglial activation correlates with amyloid burden [22]. Furthermore, there appears to be an initial increase in microglial activation early in the disease that is sustained, even if plaque burden decreases, suggesting chronic activation of microglia [23].

Epidemiological studies have found that long-term, high-dose non-steroidal anti-inflammatory use reduced an individual’s risk for AD [24]. However, interventional as well as preventative trials have failed to show significant treatment effects [25, 26]. The exact involvement of immune activation in AD pathogenesis, whether a cause or consequence, is still somewhat controversial. However, it has been shown that inflammation can drive AD pathogenesis once it has begun. Several studies have shown that increasing inflammation in models of amyloid deposition is beneficial while increasing inflammation in tauopathy models is detrimental [7, 27–29].

In an acute model, Lee et al. [29] stimulated microglia by injecting the endotoxin lipopolysaccharide (LPS) into the hippocampus of 4.5-month-old rTg4510 mice, a tauopathy model, and observed significant increases in pre-tangle phospho-tau 1 week after administration. In a chronic model of inflammation, hTau mice lacking the fractalkine receptor (CX3CR1) exhibited not only significantly greater pathology but also a significantly earlier onset of pathology and cognitive impairments. Furthermore, they observed in CX3CR1-deficient mice, systemic administration of LPS was sufficient to cause phosphorylation of endogenous tau at the paired helical filament-associated sites AT8 and AT180 [11]. These studies highlight the ability of pro-inflammatory activation of microglia to exacerbate, and even initiate, tau pathology.

Recently, genetic risk-factors for AD have been identified that are expressed uniquely in the innate immune system, implicating microglial involvement in AD susceptibility and/or pathogenesis [30–34]. Elevations in adaptive and innate immune markers have been widely reported in AD—in both animal models and human subjects—and correlate with disease progression (reviewed in [35, 36]). Polymorphism associations of IL-1 and TNF-α have been observed in AD patients [37, 38]. Genome-wide association studies have identified more than 20 gene variants associated with an increased risk of late onset AD (LOAD), including CR1, CD33, MS4A, CLU, and HLA-DRB5 (reviewed in [39, 40]). ApoE4 and, more recently, triggering receptor on myeloid cells (TREM) 2, were identified as conferring an increased risk of LOAD [41]. It has been proposed that overproduction of ApoE by activated glia might exacerbate inflammation, as ApoE4 stimulated IL-1 at significantly lower concentrations than ApoE3 [42]. The discovery of these innate immunity genes as risk factors for AD further strengthens the link between immune activation and AD pathogenesis.

The identification of immune-related gene risk factors for AD further implicates involvement of the immune system in disease etiology. It may be the case that immune activation and inflammation play a dual role in AD, depending on the stage of the disease. Of the risk-associated genes discussed, all have been studied in the context of amyloid deposition and have shown that a reduction in phagocytic capacity is associated with increased amyloid burden. However, both TREM2 and CR1 have opposing effects on amyloid and tau pathology, in keeping with the body of work indicating that activation of microglia is beneficial for amyloid pathology while detrimental to tau pathology. However, microglial dysfunction may also play a role in disease progression.

There is evidence that microglial function is impaired in age and AD. Hickman et al. [43] observed reduced gene expression of amyloid beta-binding proteins, as well as reduced expression of amyloid-degrading enzymes. Furthermore, stimulation of a microglial cell line, with tumor necrosis factor (TNF) α recapitulated this phenotype, suggesting that sustained exposure to pro-inflammatory cytokines may inhibit microglial function. Recently, beclin 1 was implicated as a regulator of phagocytosis and was found to be impaired in human microglia isolated from late-stage AD cases [44]. Given the involvement of microglia in maintenance of CNS homeostasis and learning and memory, restoration of normal microglial function by modulating the inflammatory milieu may be a potential therapeutic target for AD [45].

The neuroinflammatory hypothesis, that links amyloid deposition to tau pathology via immune activation, has support from human tissue, mouse models, and genetic risk factors for AD. It appears as though microglia become activated initially and may clear amyloid. However, as the disease progresses the ability of microglia to phagocytose amyloid reduces and this once protective immune activation turns neurotoxic, directly causing neurodegeneration and either leading to or exacerbating tau pathology. Since the failure of clinical trials using non-steroidal anti-inflammatory drugs to improve cognitive outcomes or reduce conversion to AD, attention has turned to immunomodulators as potential therapeutics. These compounds, such as CD200, CD22, and CX3CL1, modulate, rather than suppress, microglial activation.

### CX3CL1/CX3CR1

**Fig. 1:**
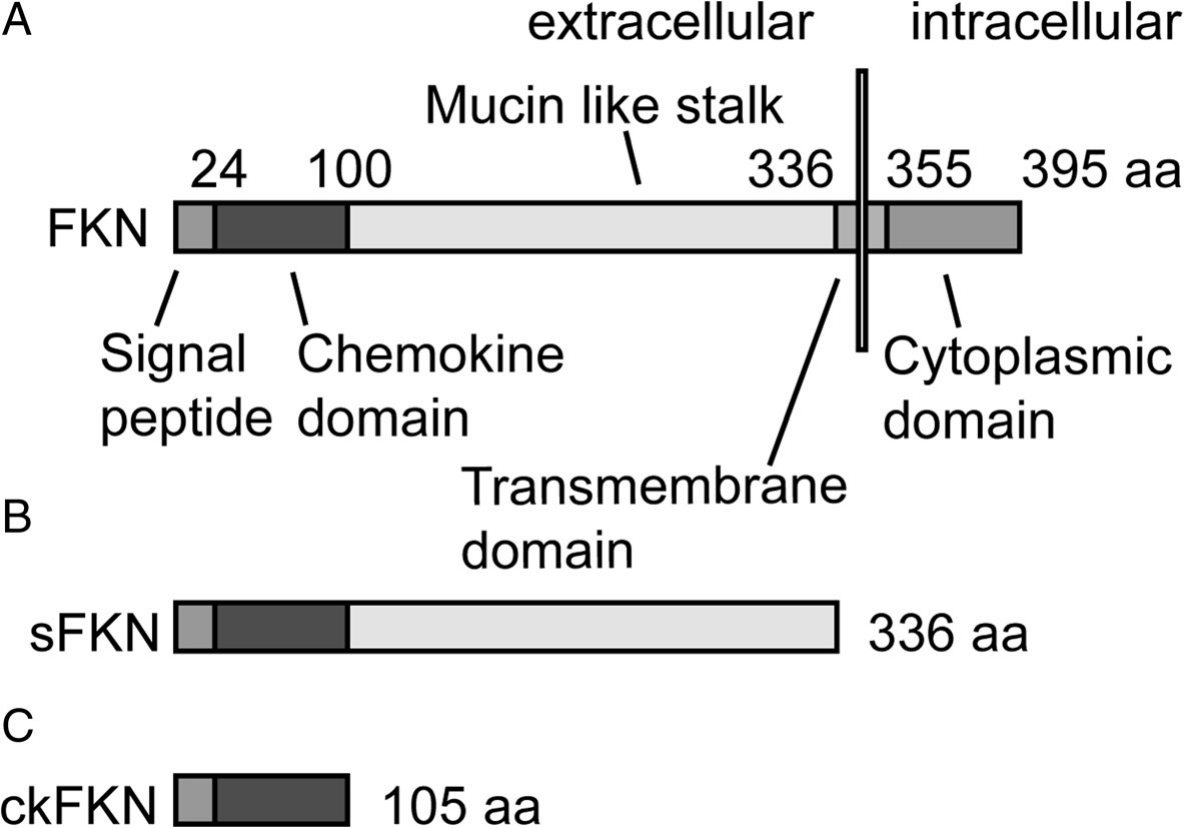
Diagrammatic representation of FKN and proteolytic cleavage fragments. **a** Full-length FKN variant is membrane-associated protein with a short cytoplasmic domain, a single transmembrane domain, and an extracellular mucin-like stalk and chemokine domain. **b** Soluble FKN (sFKN). Putative ADAM10/17 cleavage variant, generating a soluble FKN that includes the mucin-like stalk. **c** A theoretical fragment of FKN containing only the chemokine domain. This peptide is typically used for recombinant peptide studies

Fractalkine is the only member of the CX3C chemokine family and, unlike many other chemokines, shares a one-to-one relationship with its cognate receptor, CX3CR1 [46, 47]. CX3CR1 is a G protein-coupled receptor (GPCR) that signals through the Gq pathway, in turn activating PI-3 kinase pathway [48]. FKN is expressed in neurons and peripheral endothelial and its receptor is produced by myeloid cells (including microglia), T cells, and natural killer cells [49, 50]. FKN is a type I transmembrane protein with an N-terminal chemokine domain attached to a long, highly glycosylated mucin-like stalk with a short membrane-spanning domain and intracellular domain. FKN can be cleaved by a disintegrin and metalloprotease (ADAM) 10, ADAM 17, and cathepsin S to generate a soluble fragment [2–4] (Fig. 1). Both the membrane-bound and soluble forms of FKN are capable of signaling through CX3CR1, although there may be differences in affinity and biological activity between these two ligands [1].

In the periphery, membrane-bound FKN can promote adhesion of monocytes to endothelial cells, and the soluble ligand can cause chemotaxis of monocytes, T cells, and natural killer cells [51, 52]. Genetic ablation of FKN causes reduced monocyte survival and reduced ability of CX3CR1^+^ macrophages to sense the gut lumen [53]. Expression of the chemokine domain of FKN was sufficient to rescue survival of certain populations of monocytes and to rescue the ability of CX3CR1^+^ macrophages to form transepithelial dendrites. In the CNS, fractalkine is predominantly expressed by neurons, and CX3CR1 is found only on microglia [1, 12, 50].

Single-nucleotide polymorphisms (SNPs) of *CX3CR1* have been implicated as risk factors in several diseases. There is a correlation between these two SNPs and age-related macular degeneration and coronary artery disease [54–57]. These SNPs increase risk for age-related macular degeneration but reduce the risk of coronary artery disease. It was also observed that HIV-positive patients homozygous for both SNPs more rapidly converted to AIDs [58]. These SNPs may impact receptor function or receptor expression on monocytes. There is evidence that these SNPs reduce affinity of the receptor for FKN and may reduce surface receptor expression on monocytes [55]. Genetic knockout of both the receptor and the ligand have shed light on the role of FKN signaling in development, homeostasis, and disease.

#### CX3CR1 knock out models and neurodegenerative diseases

Generation of a reporter mouse, replacing endogenous *Cx3cr1* with a green fluorescent protein (GFP) reporter under control of the *Cx3cr1* promoter, demonstrated that CX3CR1 expression is restricted to microglia in the CNS [12, 59]. This reporter line has been used extensively to investigate the impacts of disrupting FKN signaling from the developing to the aged brain. In development, CX3CR1 deficient mice were observed to have delayed synaptic pruning. Microglial number was transiently reduced in *Cx3cr1−/−* mice compared to wild-type controls while the number of dendritic spines was increased. This indicates that CX3CR1 deficiency may delay maturation of synapses by delaying engulfment during development [60]. In the adult brain, CX3CR1 has been identified as necessary for layer V cortical neuron survival [61]. These findings implicated FKN signaling in normal brain development and homeostasis but ablation of CX3CR1 also affects cognition.

Disruption of FKN signaling has deleterious effects on both neurogenesis and cognition. *Cx3cr1−/−* mice were found to have a significant reduction in neurogenesis, which could be reversed by IL-1 receptor antagonist administration [62]. When behaviorally assessed, these mice were found to have motor learning impairments, spatial recall impairments, and fear-associated recall impairments. These cognitive impairments were associated with reduced synaptic plasticity. Antagonism of IL-1β signaling successfully reversed hippocampal-dependent learning but not motor learning [63].

Disruption of FKN signaling has also been studied in the context of neurodegenerative disorders. In a seminal paper, Cardona et al. [12] described the impact of CX3CR1 knock out in several models of neurodegeneration. Broadly, they found that microglia lacking CX3CR1 are cytotoxic in models of systemic inflammation, amyotrophic lateral sclerosis (ALS), and Parkinson’s disease (PD). Mice lacking CX3CR1 were more susceptible to neuron loss upon systemic injection of LPS. Furthermore, adoptive transfer of *Cx3cr1−/−* microglia from LPS-challenged mice into wild-type mice also produced neurotoxicity in the area of the injection site. Interestingly, microglia lacking CX3CR1 did not migrate away from the injection site whereas microglia with intact CX3CR1 signaling migrated from the injection site. Inhibition of IL-1β signaling blocked the neurotoxic effects of and restored the migratory capacity of *Cx3cr1−/−* microglia. Similarly, in the SOD^G93A^ mouse model of ALS, disruption of FKN signaling caused greater neuron loss, reduced hindlimb strength, and shortened lifespan. In a toxic model of PD, loss of FKN signaling by knocking out either the receptor or the ligand resulted in significantly greater neurotoxicity [12].

In models of ischemia, the role of FKN has been less straightforward. It has been shown that genetic ablation of either CX3CR1 or CX3CL1 reduced infarct size in ischemic models [8, 10, 64, 65]. However, administration of exogenous CX3CL1 into wild-type rats subjected to middle cerebral artery occlusion was found to be beneficial, reducing infarct volume and improving behavioral outcomes [64]. These effects were not seen when exogenous CX3CL1 was administered to *Cx3cl1−/−* mice, in which exogenous CX3CL1 ablated the protective effects observed in untreated *Cx3cl1−/−* mice. Cipriani et al. [64] attributed these seemingly contradictory results to differential response of wild-type primary microglia to exogenous CX3CL1 in vitro. They observed no reduction in TNFα secreted from stimulated wild-type microglia after treatment with CX3CL1 whereas microglia isolated from *Cx3cl1−/−* mice did reduce TNFα secretion after treatment with CX3CL1.

FKN signaling has been studied in spinal cord and brain injury models. In a mild traumatic brain injury model, FKN signaling has been shown to have a temporally dependent action. In the first 15 days after the injury, *Cx3cr1−/−* mice exhibit significantly less impairment as well as reduce pro-inflammatory markers. However, 30 days after injury *Cx3cr1−/−* mice have significantly greater memory impairment on the Morris water maze as well as significantly elevated pro-inflammatory markers compared to wild-type controls [66]. Similar results as those found in stroke models have been observed in a spinal cord injury model, that is, disrupting FKN signaling confers neuroprotection and improves behavioral outcomes [67]. However, these effects were largely attributed to a blunting of the inflammatory phenotype acquired by infiltrating monocytes.

#### CX3CR1CX3CL1 in AD

The effects of disrupting FKN agonism has also been studied in AD models. FKN expression in both the hippocampus and cortex is reduced in AD brain compared with non-demented controls [13], suggesting a dysregulation of this pathway in AD. Several studies have shed light on the role of FKN signaling in AD. It has been shown in two models of amyloid deposition that disrupting FKN signaling by knocking out *Cx3cr1* is beneficial. In both models, a reduction in amyloid plaques was observed accompanied by an increase in microglial phagocytosis [7, 68]. Conversely, it has been observed that FKN signaling is beneficial in tau pathology. In the hTau mouse model of tauopathy, it was observed that disrupting FKN signaling worsened pathology and accelerated insoluble tau deposition. These increases in pathology were accompanied by an impairment in spatial working memory [11]. A further study showed deficits in Morris water maze and, importantly, that CD45 immunoreactivity precedes tau hyperphosphorylation in this model. In an adoptive transfer model, wild-type mice receiving microglia from hTau *Cx3cr1−/−* mice showed increased AT8 immunoreactivity that could be blocked with IL-1Ra [69]. Additional studies in amyloid models have also observed a worsening of tau pathology in the absence of FKN signaling [13, 16]. Thus, FKN may have complex interactions with the hallmark pathologies of AD and may be neuroprotective or neurotoxic at different timepoints in disease progression.

Interestingly, recent reports implicate direct interaction between CX3CR1 and tau. Bolos et al. (2017) [70] demonstrated that tau can directly bind to CX3CR1 and competes with FKN. This may result in a disruption of the neuronal/glial communication and thus uncoupling of microglial activation. Bolos et al. (2017) [70] also report a significant increase in immunohistochemical staining for FKN in the AD brain, however, more recently, they reported a concomitant reduction in soluble FKN in the cerebrospinal fluid of AD patients [71] compared to non-demented age-matched controls. Suggesting that there may be a reduction in FKN processing to the soluble form. The soluble form of FKN has been suggested to be more anti-inflammatory and neuroprotective than the membrane bound form [72]. As tau can directly stimulate expression of pro-inflammatory cytokines in primary microglia [73], these data indicate both a reduction in anti-inflammatory signaling, by reduced cleavage of FKN, synergizes with the pro-inflammatory stimulus of extracellular tau to contribute to neuroinflammation in AD.

#### CX3CL1 gain of function studies

A series of studies have examined the effects of over expression of FKN in different neurological disorders. Studies increasing FKN in PD have found that increased CX3CR1 agonism is beneficial. Bachstetter et al. [74] described the protective effects of a soluble FKN ligand in the 6-hydroxydopamine model of PD. In this model, increased FKN agonism resulted in reduced microglial activation, reduced lesion size, and protection of dopaminergic neurons in the substantia nigra. Similar results were observed when *Cx3cl1−/−* mice were lesioned with MPTP and injected with adeno-associated virus (AAV) over-expressing FKN. Animals injected with FKN showed significant improvement compared to AAV over-expressing GFP-injected mice [72]. Finally, it was shown that FKN was also able to halt neurodegeneration in a viral model of α-synuclein over expression [75]. These data suggest an increase in CX3CR1 agonism is a therapeutic target for the treatment of neurodegeneration in PD.

Examining the effects of FKN over expression on tauopathy has yielded encouraging results. Over expression of a soluble FKN (sFKN; predicted to be the ADAM10/17 cleavage product, Fig. 1) via AAV in the rTg4510 mouse model of tauopathy reduced both soluble and insoluble phospho-tau pathology, ameliorated neuron loss, and reduced microglial activation. This is one of the first therapeutic interventions that were able to reduce brain atrophy in this model. Interestingly, from the *Cx3cr1−/−* studies described above, one may predict that the FKN over expression may worsen amyloid pathology [7, 9, 68]. However, when sFKN was over expressed in the APP/PS1 model of amyloid deposition, there was not a significant impact on amyloid pathology [76]. This highlights that CX3CR1 agonism may be a potential target for immunomodulation, with beneficial effects on tauopathy and no observed detrimental effects on amyloid deposition.

#### Fractalkine signaling: does the ligand matter?

There is some evidence that different fragments of the FKN ligand may have different functional outcomes. Fractalkine signaling has been implicated in neuropathic pain in spinal injury models [77–79]. In these studies, neuropathic pain in injury models was alleviated by antibody antagonization of CX3CR1, and in non-injured rats neuropathic pain was induced by activation of CX3CR1 with intrathecal injections of exogenous fractalkine peptide comprising just the chemokine domain. Furthermore, Clark and Malcangio [15] show that infusion of this chemokine peptide of FKN (ckFKN, Fig. 1) elicits mechanical allodynia, while infusion of sFKN (which contains the full mucin-like stalk, Fig. 1) does not. They also demonstrate a difference in calcium mobilization upon binding between these two fragments. Interestingly, it has been identified that cathepsin S is necessary for the production of pain-inducing FKN in a spinal injury model [2, 77]. In silico analysis of substrate preference of cathepsin S indicates membrane-bound FKN as a potential substrate [77, 80–82] and a recent study published that cathepsin S cleaves membrane bound FKN to generate a soluble fragment that migrates at a lower apparent molecular weight than the ADAM10/17 cleavage product [83]. These data suggest that removal of the mucin-like stalk (either partially or in whole) may alter the microglial response to FKN, which can elicit different effects and/or microglial phenotypes depending on the identity of the soluble ligand.

Differences in signaling also appear to occur in neurodegenerative diseases. In Parkinson’s disease, studies have shown that a soluble FKN is necessary for neuroprotection in both a MPTP and an α-synuclein over expression model, whereas a membrane bound form of FKN had no protective effects [72, 75]. These data indicate that neuroprotective signaling in the CNS by FKN is dependent on its cleavage. The results in Alzheimer’s disease are not as clear. In order to determine the relative contributions of soluble versus membrane-associated FKN to AD pathology, a transgenic mouse that only expresses the chemokine domain of FKN, termed SolTg or CX3CL1^105Δ^, was developed and crossed with AD mouse models. In a cross of this ckFKN-expressing mouse with an APP/PS1; *Cx3cl1−/−* mouse, Lee et al. [16] observed that ckKFN failed to have an impact on either amyloid or tau pathology as compared to APP/PS1; *Cx3cl1−/−* control mice. They argued that it is therefore the membrane-bound variant of FKN, and not a soluble ligand, that impacts amyloid pathology. In a more recent report, Bemiller et al. [14] observed an increased susceptibility to LPS-induced tauopathy and microglial activation in the mice expressing only ckFKN. Furthermore, they show that hTau mice expressing ckFKN (hTau/CX3CL1^105Δ^) had similar phospho-tau pathology and cognitive deficits as hTau; *Cx3cl1−/−* mice, both of which had significantly greater phospho-tau pathology and cognitive deficits than hTau mice expressing endogenous FKN. The authors observed a reduction in CX3CR1 receptor expression on microglia in ckFKN expressing mice as compared to either non-transgenic or *Cx3cl1−/−* mice, perhaps explaining the reduced biological activity of ckFKN as compared to endogenous FKN in this model. Contrary to these data, Nash et al. [76] observed neuroprotective effects using a different soluble FKN, the putative ADAM10/17 cleavage product, which contains the mucin-like stalk and the chemokine domain. They observed reductions in brain atrophy, tau pathology, and neurodegeneration in the rTg4510 tau model [76]. These data may indicate that the form of the soluble fragment (with or without the mucin stalk) affects how the chemokine signals in these neurodegenerative models and that proteolytic cleavage could be a mechanism for regulation of fractalkine activity in vivo. More research is needed to determine if there are differential effects of these soluble FKN variants and how this could regulate neuroinflammation.

## Conclusion

Recent discovery of innate immunity genes conferring increased risk of AD has sparked greater interest in the role of inflammation in AD. As our understanding of the role inflammation plays in AD increases, it becomes progressively clear that immunomodulators are a more attractive therapeutic approach rather than broadly suppressing immune activity. One such target is fractalkine, which shares a one-to-one relationship with its receptor. In the CNS, fractalkine is expressed by neurons and signals its receptor by microglia, thus allowing neurons to directly influence neuroinflammation. Fractalkine’s involvement in neurodegeneration has been relatively controversial, with disruption of fractalkine signaling being beneficial in some disease states (amyloid pathology and stroke) and yet detrimental in other neurodegenerative diseases (PD, ALS, and tauopathies). Increasing FKN agonism has been shown to be neuroprotective in both AD and PD models; however, there is some controversy in the field regarding which form of FKN mediates the observed neuroprotection. More research is needed to fully understand the therapeutic potential of FKN in AD, with special attention to ligand processing and how this regulates microglial activation.

## Abbreviations

ADAM: A disintegrin and metalloprotease
AAV: Adeno-associated virus
AD: Alzheimer’s disease
Aβ: Amyloid-β
APP: Amyloid precursor protein
ALS: Amyotrophic lateral sclerosis
CNS: Central nervous system
CX3CL1; FKN: Fractalkine
GPCR: G protein-coupled receptor
GFP: Green fluorescent protein
IL: Interleukin
LPS: Lipopolysaccharide
LOAD: Late onset Alzheimer’s disease
TSPO: Microglial translocator protein
MAPT: Microtubule associated protein tau
NFTs: Neurofibrillary tangles
PD: Parkinson’s disease
SNPs: Single-nucleotide polymorphisms
TREM2: Triggering receptor expressed on myeloid cells 2
TNFα: Tumor necrosis factor-α
